# Morpho-Functional Effect of a New Collagen-Based Medical Device on Human Gingival Fibroblasts: An In Vitro Study

**DOI:** 10.3390/biomedicines11030786

**Published:** 2023-03-05

**Authors:** Tea Romasco, Pier Michele Mandrillo, Erica Morsut, Margherita Tumedei, Domitilla Mandatori, Morena Petrini, Maria Cristina Curia, Francesco De Angelis, Camillo D’Arcangelo, Adriano Piattelli, Natalia Di Pietro

**Affiliations:** 1 Department of Medical, Oral and Biotechnological Sciences, “G. d’Annunzio” University of Chieti-Pescara, 66100 Chieti, Italy; 2 Center for Advanced Studies and Technology-CAST, “G. d’Annunzio” University of Chieti-Pescara, 66100 Chieti, Italy; 3 Independent Researcher, 38121 Taranto, Italy; 4 Independent Researcher, Guna S.p.a., 20132 Milan, Italy; 5 Department of Medical, Surgical, and Dental Sciences, University of Milan, 20122 Milan, Italy; 6 Maxillo-Facial Surgery and Dental Unit, Fondazione IRCCS Ca’ Granda Ospedale Maggiore Policlinico, 20122 Milan, Italy; 7 School of Dentistry, Saint Camillus International University of Health and Medical Sciences, 00131 Rome, Italy; 8 Facultad de Medicina, UCAM Universidad Católica San Antonio de Murcia, Guadalupe, 30107 Murcia, Spain

**Keywords:** collagen, FAK, gingival fibroblasts, Hippo pathway, mechanotransduction, oral soft tissue regeneration, parodontology, regenerative medicine, wound healing, YAP/TAZ

## Abstract

Maintaining periodontal and peri-implant soft tissues health is crucial for the long-term health of teeth and dental implants. New biomedical strategies aimed at avoiding connective tissue alterations and related diseases (e.g., periodontitis and peri-implantitis) are constantly evolving. Among these, collagen-based medical products have proven to be safe and effective. Accordingly, the aim of the present study was to evaluate the effects of Dental SKIN BioRegulation (Guna S.p.a., Milan, Italy), a new injectable medical device composed of type I collagen of porcine origin, on primary cultures of human gingival fibroblasts (hGF). To this end, hGF were cultured on collagen-coated (COL, 100 µg/2 mL) or uncoated plates (CTRL) before evaluating cell viability (24 h, 48 h, 72 h, and 7 d), wound healing properties (3 h, 6 h, 12 h, 24 h, and 48 h), and the activation of mechanotransduction markers, such as FAK, YAP, and TAZ (48 h). The results proved a significant increase in cell viability at 48 h (*p* < 0.05) and wound closure at 24 h (*p* < 0.001) of hGF grown on COL, with an increasing trend at all time-points. Furthermore, COL significantly induced the expression of FAK and YAP/TAZ (*p* < 0.05), thereby promoting the activation of mechanotransduction signaling pathways. Overall, these data suggest that COL, acting as a mechanical bio-scaffold, could represent a useful treatment for gingival rejuvenation and may possibly help in the resolution of oral pathologies.

## 1. Introduction

It is well established that periodontal and peri-implant soft tissues play a crucial role in maintaining the long-term health of teeth and dental implants [1]. Indeed, besides the aesthetic contribution, a well-organized connective tissue creates a seal that guarantees mechanical and biological protection of the underlying bone [1,2].

As a matter of fact, in concomitance with the presence of a diseased periodontal tissue or with the insertion of dental implants, the connective tissue undergoes significant alterations, provoking the lowering of such defense mechanism that, in association with an increase in inflammation and bacterial infiltration, promotes the onset of periodontitis and peri-implantitis [3,4].

Furthermore, the lack of an appropriate keratinized mucosa and a buccal depth may also play an important role in the development and degeneration of periodontal and peri-implant inflammation [5,6,7]. These deficiencies are typically related to increased gingival and plaque indices, probing depths, attachment levels, bleeding on probing, mucosal recession, marginal bone levels, and discomfort when brushing [8,9,10,11,12,13].

Such complications require the rapid activation of healing procedures in order to allow the restoration of soft tissues structure and function. This specific response, as in wound healing, may involve three temporally overlapping but distinct phases: (i) blood clotting and inflammation; (ii) new tissue formation; (iii) tissue remodeling [14,15]. In general, the healing process of connective tissues requires the involvement of different factors and resident cell populations capable of synthesizing structurally appropriate extracellular matrices, which in turn can support cell activation, proliferation, and differentiation [15].

In particular, gingival fibroblasts play a significant role in the formation and remodeling of periodontal and peri-implant tissues [16]. They synthesize and organize the extracellular matrix (ECM), providing structural integrity to the tissues and creating a network of collagen fibers, which allows the re-stabilization of the mucosal seal [2]. Moreover, fibroblasts are also able to communicate with other cells by secreting growth factors and cytokines or by releasing them in the ECM, thereby creating complex cellular communication networks and transmitting mechanosensory signals [17].

In this regard, it has been shown that in cultured fibroblasts, focal adhesions are one of the most widely described adhesive structures [18]. They allow cells to perceive the mechanical properties of the matrix, representing an important determinant of tissue homeostasis [19]. After integrin-mediated binding of cells to the ECM, Focal Adhesion Kinase (FAK) is temporally one of the first molecules to be activated and recruited into adhesions, where it undergoes autophosphorylation [20]. FAK provides critical control of the cell processes involved in adherence-dependent signaling [21], regulating cell migration, proliferation, and survival [22]. FAK is also involved in sensing the mechanical properties of the ECM, a process that contributes to the regulation of cell migration through the intracellular signaling of the mechanical message.

In addition to that, other mechanotransduction mechanisms may be involved, including the regulation of the Hippo pathway, whose terminal purpose is to limit the transcriptional activity of anti-Phospho-WW domain-containing transcription regulator protein 1 (WWTR1, TAZ) and anti-Yes-associated protein (YAP) by preventing YAP/TAZ from entering the nucleus. For that reason, the latter are considered key mechanotransducers, so they are able to sense and transduce mechanical cues, such as the stiffness of the extracellular environment [23]. In fact, it has been demonstrated that when a single cell stretches on the ECM, the cytoskeleton adaptation to the cell shape causes YAP/TAZ activation and nuclear accumulation, promoting cell proliferation and inhibiting differentiation [24]. Conversely, when cell morphology is manipulated into a round and compact shape (limiting adhesion to a very small ECM adhesive area), YAP/TAZ are excluded from the nucleus through phosphorylation and their transcriptional properties are disabled, resulting in the initiation of differentiation and the interruption of proliferation [24].

Several upstream stimuli, such as mechanical cues from the cytoskeleton, cellular stress, cell-cell adhesion, cell polarity, and extracellular signaling, result in the activation of the Hippo pathway. In fact, when cells are plated on a very soft substratum, the core kinases MST1/2 form a heterodimer with the adaptor protein SAV1, which, in turn, activates the LATS1/2 kinases. The activated LATS1/2-MOB1A/B complex induces YAP/TAZ phosphorylation at S127/S89, respectively. Consequently, nuclear exclusion of YAP/TAZ occurs, keeping them in an inactive status and preventing the transcription through TEA domain family members 1–4 (TEAD1–4). On the contrary, when a stiff matrix is present, the Hippo pathway is inactive, unphosphorylated YAP/TAZ move to the nucleus and interact with TEAD to promote cell proliferation and migration [23].

In addition to this, since the main function of gingival fibroblasts is the synthesis of important components of the ECM, for example collagen, which dynamically interact with the newly formed collagen matrix to generate structural support to the tissue, it is clear that the collagen itself can act as a stimulus for proliferation, differentiation, and activation of these cells [25]. Various sources and formats of collagen have been proposed for periodontal and peri-implant tissues regeneration. As an example, due to their advantageous properties, such as good biocompatibility, hemostasis support, low antigenicity, degradation by specific enzymes, and chemotactic attraction of regenerative cells, collagen barrier membranes have been used for Guided Bone Regeneration (GBR) and Guided Tissue Regeneration (GTR) procedures for several years [26]. Moreover, collagen matrices are generally used for soft tissue regeneration, collagen sponges and cones for the stabilization of oral wounds and extraction sockets, and along with aesthetic medicine development, collagen is also used in its injectable form as a tissue stimulator [27,28].

In this context, one of the main objectives of dental research is to provide innovative treatments aimed at restoring and preserving oral, perioral, and peri-implant soft tissues. The most advanced therapies in regenerative medicine use gene editing, stem cells, and tissues to handle a wide range of conditions. One of their purposes is to stimulate the endogenous repair of tissues by manipulating stem cells or optimizing the intrinsic characteristics and plasticity of differentiated cells in adult tissues. In this regard, fibroblasts emerge as an alternative source to stem cells since they express phenotypic and regenerative characteristics. Interestingly, fibroblasts of the oral cavity have shown improved regenerative capacity in comparison with other fibroblast populations. In addition, thanks to their easy access by means of minimally invasive procedures without generating aesthetic problems, their easy and rapid in vitro expansion, and their great ability to respond to extrinsic factors, oral fibroblasts represent an attractive and interesting resource for regenerative medicine [25].

Although there is already evidence of the regenerative potential of human Gingival Fibroblasts (hGF) in ECM synthesis, wound healing, mechanotransduction, and immunological regulation [20,25,29,30], nowadays it is not fully exploited as an in vitro model to deepen the knowledge about these phenomena or as an effective means to validate different products or medical devices implied in tissue regeneration.

Hence, the aim of this study was to investigate the effect of Dental SKIN BioRegulation (Guna S.p.a., Milan, Italy), an injectable medical device containing type I porcine collagen as the main component, on cultures of primary hGF. Indeed, collagen of porcine origin is already widely used in different clinical fields [31,32,33], including dentistry [26,34,35], because of its high similarity to human collagen and therefore, its excellent biocompatibility. The main focus of this research was to study the effect exerted by a coating of this injectable collagen on cell viability, proliferation, and cell migration for wound healing, as well as on the expression and activation of some mechanotransduction markers. Modulating ECM-cell interactions and the mechanotransduction signaling pathway are the ideal targets of advanced therapies to induce tissue regeneration. The plasticity and adhesion property of hGF is evident when the substrate topography and roughness are manipulated, so as to induce the expression of different cell phenotypes. This explains why fibroblasts growing on soft surfaces develop a strong network of actin fibers with an elongated morphology, fibronectin synthesis, and a decreased phosphorylation of time-dependent FAK to become more mechanically stable [36]. Conversely, a rough surface creates a pattern of mechanical stress that induces a higher density of focal contacts and a different expression of ECM molecules [37]. Therefore, an effective surface for tissue engineering, in addition to provide chemical, biological, and mechanical signals, should also provide adequate topography, rigidity, and functionalization of the scaffolds to optimize the intrinsic regenerative characteristics of the cell population [38].

Interestingly, Dental SKIN BioRegulation (Guna S.p.a., Milan, Italy) has been designed to help counteract the physiological aging of connective tissues and intended to act as a mechanical bio-scaffold, providing physical and chemical support to collagen fibers present in the soft tissues around teeth. In particular, following in the footsteps of the present study design, it was previously demonstrated that this medical device,,though mentioned with another commercial name and tested on specialized fibroblasts in tendon connective tissue (human tenocytes), not only induced type I collagen secretion and increased the levels of Tissue Inhibitor of Matrix Metalloproteinases (TIMP-1), suggesting an anti-collagenolytic effect, but also upregulated the expression of focal adhesions and mechanosensors (FAK, YAP/TAZ), implying a mechanical input for cell migration [39,40]. Clinical studies also corroborated these data, demonstrating that the collagen-based medical compound was effective in connective tissue repair, even in osteoarticular pathologies [41,42,43,44].

Thus, since the biological effects triggered by Dental SKIN BioRegulation (Guna S.p.a., Milan, Italy) in hGF have never been investigated before, the results of the present study could shed light on the mechanisms underlying the potential beneficial effects of its use in the dental field and could represent a potential approach to support collagen maturation and trigger soft tissue healing around natural teeth or dental implants. In this way, the null hypothesis assumes that the collagen coating could not affect or show significant differences in cell viability and proliferation, cell migration, and mehanotransduction properties of hGF compared to control cultures seeded on uncoated plastic substrates.

## 2. Materials and Methods

### 2.1. Materials

Collagenase/dispase (cat. 10269638001), phosphate-buffered saline (PBS, cat. D8662), Dulbecco’s modified Eagle medium low glucose (DMEM, cat. D6046), 0.5% trypsin/0.2% ethylenediaminetetraacetic acid (EDTA) solution (cat. 59418C), L-glutamine (L-Glu, cat. G7513), penicillin-streptomycin (P/S, cat. P4333), Amphotericin B solution (cat. A2942), Dimethyl sulfoxide (DMSO, cat. D2438), hexamethyldisilazane (HMDS, cat. 804324), sodium cacodylate trihydrate (NaCaCO, cat. C0250), glutaraldehyde solution (cat. 49630), ethyl alcohol (cat. 1009832511), and osmium tetroxide solution (OsO_4_, cat. 75632) were purchased from Sigma-Aldrich (Merk Life Science S.r.l., Milan, Italy), whereas fetal bovine serum (FBS, cat. 41A0045K) was from Life Technologies (Thermo Fisher Scientific, Waltham, MA, USA). Anti-Focal adhesion kinase (FAK, cat. A32309), anti-Phospho-WW domain-containing transcription regulator protein 1 (WWTR1, TAZ, cat. E5P2N), and anti-Yes-associated protein (YAP, cat. A32371) primary antibodies were from Antibodies.com (Antibodies.com Europe AB, Stockholm, Sweden), whereas anti-Phospho-YAP (pYAP, Ser127, cat. BK130085) primary antibody was from Cell Signaling (Cell Signaling Technology, Milan, Italy). CD105 (FITC-conjugated antibody, cat. 326-040), CD73 (PE-conjugated antibody, cat. 550257), CD90 (FITC-conjugated antibody, cat. 555595), CD326 (PerCP-Cy5.5-conjugated antibody, cat. 347199), CD45 (FITC-conjugated antibody, cat. 196-040), besides BD™ FACS™ Lysing Solution (FIX, cat. 349202) and BD FACS™ Permeabilizing Solution (PERM, cat. 340973) were purchased from Becton Dickinsons (BD Biosciences, San Jose, USA), instead Calcein AM (cat. C3099) molecular probe, anti-pWWTR1 (pTAZ, Ser89, cat. SAB5701813) primary antibody, Alexa Fluor 488- (cat. 11034), and Alexa Fluor 647- (cat. A-21235) conjugated antibodies were purchased from Invitrogen (Thermo Fisher Scientific, Waltham, MA, USA).

### 2.2. Collagen Coating with Dental SKIN BioRegulation

Dental SKIN BioRegulation (100 µg/2 mL sterile vial) was kindly provided by Guna S.p.a. (Guna S.p.a., Milan, Italy). This is an injectable medical device composed of collagen type I of porcine origin as the main constituent and excipients such as ascorbic acid, magnesium gluconate, pyridoxine hydrochloride, riboflavin, thiamine hydrochloride, NaCl, and water for injection. Based on the results obtained by Randelli et al. [39,40] on human tenocytes, in this study, we hypothesized that allowing hGF to grow on a coating layer made up of this collagen compound could represent a substrate for cell cultures, contributing to their mechanical stimulation and influencing their proliferation and migration properties.

Dental SKIN BioRegulation (100 µg/2 mL, Guna S.p.a., Milan, Italy) was used to obtain a thin coating on multi-well plates or Petri dishes, adding 500 µL per 24-well plate and 5 mL per 100 mm diameter Petri dishes. In order to allow an appropriate adhesion of collagen to the plastic, multi-well cell culture plates and Petri dishes were incubated for 4 h at room temperature (RT) before removing the excess solution, and then the coated surfaces were left to dry under the laminar flux hood. Coated plastics were used immediately or stored at 4 °C, as recommended in a previous work [39].

hGF cultured on collagen-coated cell culture plastic substrates were considered as test cultures (COL) and compared to hGF seeded on uncoated cell culture plastic substrates used as untreated controls (CTRL).

### 2.3. Cell Culture

The hGF used for the 2D culture experiments were isolated from human gingival biopsies obtained from nine patients undergoing partial gingivectomy procedures at the dental clinic of the University G. D’Annunzio Chieti-Pescara (CE, N° 1968-24/07/2020). The collected gingival samples were immediately washed with a physiological solution added with 1% Amphotericin B and 1% P/S before being transferred into a solution of collagenase/dispase for enzymatic digestion for 1 h at 37 °C. Subsequently, the digested specimens were placed in a 60 mm diameter Petri dish with a culture medium composed by DMEM low glucose, supplemented with 20% FBS, 100 mM L-Glu, and 1% P/S, letting cells spontaneously migrate from the tissue. Then, the isolated hGF were grown in a controlled atmosphere (5% CO_2_ and 37 °C) until reaching the confluence and were used for all experiments. Furthermore, the evaluation of specific markers expressed by hGF was performed for their characterization: CD105 (FITC-conjugated antibody), CD73 (PE-conjugated antibody), CD90 (FITC-conjugated antibody), CD326 (PerCP-Cy5.5-conjugated antibody), and CD45 (FITC-conjugated antibody). FACSVerse, FACSDiva v 6.1.3, and IDEAS software (BD Biosciences, Milan, Italy) were used for the cytometric analysis.

Moreover, by means of Scanning Electron Microscope analysis (SEM, Phenom XL, Alfatest, Cernusco sul Naviglio, Italy), the morphology of hGF cultured on COL was compared to CTRL. In summary, the cells were cultured on 12 mm diameter round coverslips uncoated or coated with Dental SKIN BioRegulation (Guna S.p.a., Milan, Italy) into 24 well culture plates for 24 h. The samples were fixed with 3.5% glutaraldehyde in 0.1 M NaCaCO buffer for 24 h and stored at 4 °C, and then postfixed in 2% OsO_4_ prepared in the same buffer, washed in distilled water, dehydrated by consecutive incubation in increasing ethyl alcohol concentrations (50%, 70%, and 100%) and in HMDS, before being air-dried overnight at RT. After that, the dried specimens were mounted onto SEM stubs using double-sided carbon tape and sputter-coated with gold (150 Å), before acquiring images with the Phenom XL microscope.

**Figure 1:**
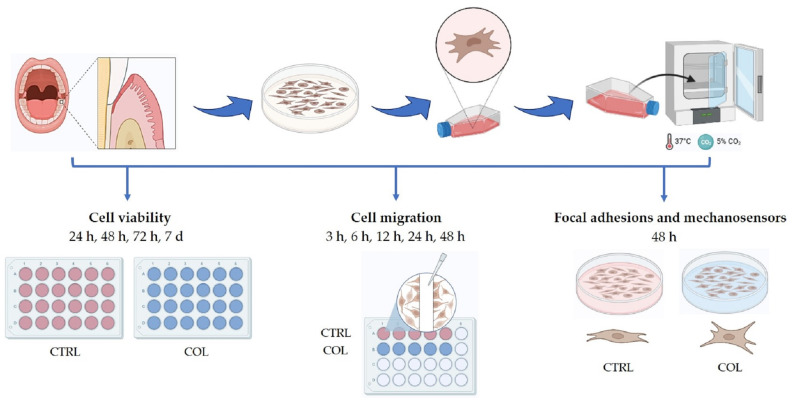
Study design summarizing the experimental conditions and treatments. CTRL: hGF cultured in standard conditions with culture medium. COL: hGF cultured in test conditions on a collagen coating with culture medium. Hours (h), days (d).

To perform morphological, functional, and molecular evaluations, cryopreserved hGF between the 3rd and the 6th passage were used. The cells were cultured in 24 well plates with culture medium (DMEM low glucose, 10% FBS, 100 mM L-Glu, and 1% P/S) for short- and long-time vitality assessments and wound-healing assay, whereas 100 mm diameter Petri dishes were used to perform flow cytometry analysis after 48 h of culture. Figure 1 summarizes the experimental design of this study.

### 2.4. Calcein AM Cell Viability Assay

For viability assessments, CTRL and COL hGF were cultured on 24 well culture plates at different cell densities for each experimental time (7 × 10^4^, 6 × 10^4^, 4 × 10^4^, 2.5 × 10^4^, and 2 × 10^4^ cells/well for 24 h, 48 h, 72 h, and 7 d, respectively). Briefly, at each experimental time-point, cells were washed twice with PBS before being trypsinized (5 mM) and harvested in PBS within flow cytometry tubes. Then, after samples centrifugation at 1500 rpm for 5 min at RT, the supernatant was removed, and all the samples, except for negative controls, were incubated for 20 min at RT in the dark with calcein AM solution (calcein AM powder dissolved in DMSO at a concentration of 1 mM) at a final concentration of 0.1 µM in PBS. Right after, the fluorescence of the samples (λex 488 nm, λem 516 nm) was measured by a FACS Canto II flow cytometer (BD Biosciences, Milan, Italy), and data were analyzed using FACSDiva v 6.1.3 and IDEAS software. Data were indicated as a mean fluorescence intensity (MFI) ratio, calculated by dividing the MFI of positive events by the MFI of negative events (MFI of secondary antibody).

### 2.5. Scratch Assay

The scratch assay is a well-known in vitro model useful for studying the wound healing properties of cells, or rather cell activation and migration in response to injury [45]. Cell migration of CTRL and COL hGF was assayed using 24 well culture plates. Cell/well were seeded at a density of 7 × 10^4^, and after 24 h of starvation (DMEM low glucose with 0.1% FBS), a scratch was made on the monolayers with a 200 µL plastic tip. Wounded monolayers were washed with DMEM low glucose to remove cell debris. Cell migration occurred in 1 mL of culture medium with 10% FBS. Scratches were observed and images were captured immediately after scratching (T0) and at each time-point after 3 h, 6 h, 12 h, 24 h, and 48 h (T3-T48), using a Personal AUtomated Lab Assistant (PAULA, Leica Mycrosystems Srl, Buccinasco, Italy) Smart Cell Imager. The percentage of wound closure (%) from T0 to T48 was determined by ImageJ software, using the following formula: [(T0 − T24)/T0] × 100 [46].

### 2.6. Flow Cytometry Analysis

The collagen coating influence on FAK, YAP/TAZ, and their phosphorylated forms protein expression by hGF was analyzed by flow cytometry analysis. After 48 h of culture, cells were detached using trypsin/EDTA solution (5 mM), collected in PBS, and centrifuged at 1500 rpm for 5 min at 4 °C. Then, cells were resuspended in FIX (1:10) and in PERM (1:10) solutions for 5 min in the dark. Subsequently, all samples, except for negative controls, were incubated with primary antibodies including anti-FAK (1:100), anti-YAP (1:50), anti-TAZ (1:50), anti-pYAP (1:200), and anti-pTAZ (1:500) for 30 min at 4 °C in the dark. After that, the same incubation parameters were used for Alexa Fluor 488- and 647-conjugated antibodies before collection of the samples in PBS for the FACS Canto II flow cytometer reading. Data were analyzed using FACSDiva v 6.1.3 and IDEAS software and expressed as mean fluorescence intensity (MFI) ratio, calculated by dividing the MFI of positive events by the MFI of negative events (MFI of secondary antibody), respectively.

### 2.7. Statistical Analysis

Data were represented as the mean ± standard error from at least three independent experiments performed using isolated cell strains from different hGF biopsies. Each experiment was conducted in technical triplicates. Statistical analysis was performed using GraphPad Prism Software Analysis version 9 (GraphPad Software, San Diego, CA, USA) via Kruskal–Wallis when comparing more than two groups or Kolmogorov–Smirnov tests to compare COL vs. CTRL. Significance was defined as a *p*-value less than 0.05.

## 3. Results

### 3.1. Analysis and Characterization of hGF and Cell Morphology

**Figure 2:**
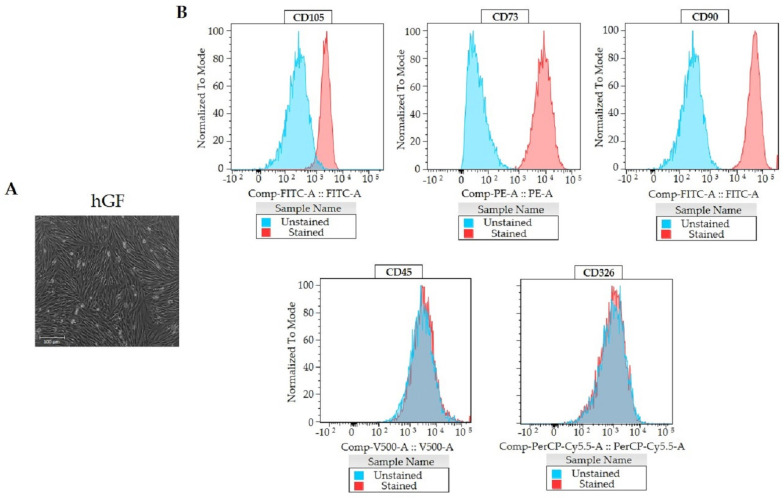
(**A**) Representative phase-contrast micrograph of isolated hGF taken by PAULA Smart Cell Imager. (**B**) Characterization of hGF. Representative graphs of CD105, CD73, CD90, CD45, and CD326 expressions evaluated by cytometric analysis.

Before verifying the collagen coating effect on our cell model, we first characterized the phenotype of hGF. Cells obtained from gingival biopsies using a combined isolation method based on enzymatic digestion and spontaneous migration [47]. These cells showed a characteristic fibroblast-like morphology (Figure 2A) and expressed typical markers: CD105, CD73, CD90, but not CD45 and the epithelial marker CD326 (Figure 2B).

**Figure 3:**
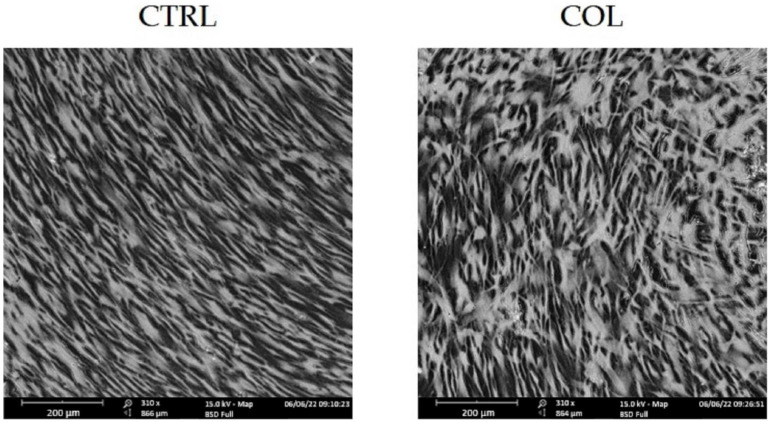
Representative SEM images of hGF cultured on uncoated (CTRL) and collagen-coated (COL) cell culture coverslips substrates. Magnification of 310x × Scale bar: 200 µm.

From the SEM observation of CTRL and COL hGF (Figure 3) after 24 h, both groups appeared with typical fibroblastoid morphology (spindle-shaped), but cells grown on the collagen coating appeared more closely intertwined with large radial filopodia and lamellipodia-like structures. On the other hand, CTRL cultures were more elongated and regularly arranged on glass, suggesting weaker attachment to the substrate.

### 3.2. Dental SKIN BioRegulation Effect on Cell Viability

**Figure 4:**
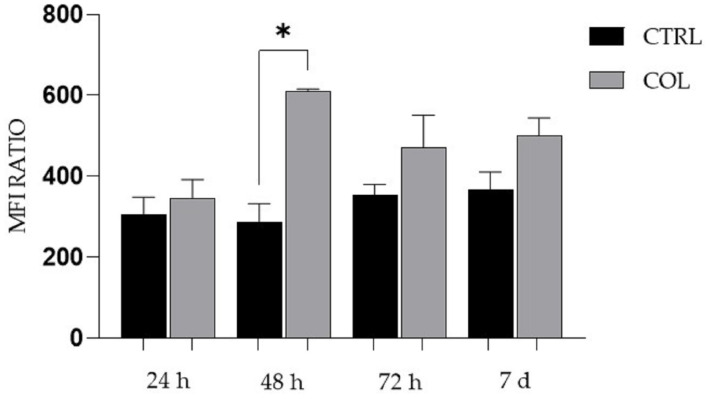
Effect of the collagen coating (COL) on hGF short- and long-term cell vitality at 24 h, 48 h, 72 h, 7 d, and 14 d, in respect to hGF on uncoated cell culture plastic substrates (CTRL). Results are shown as mean fluorescence intensity (MFI) ratio ± standard error (n ≥ 3), obtained by dividing the MFI of positive events by the MFI of negative events (MFI of secondary antibody). (* *p* < 0.05).

Based on the experimental plan reported in Figure 1, cell viability of hGF seeded on uncoated or collagen-coated cell culture substrates was assessed through cytometric analysis. As shown in Figure 4, COL hGF showed higher viability values compared to CTRL at each time point, reaching statistical significance only at 48 h.

### 3.3. Wound Healing Assay

**Figure 5:**
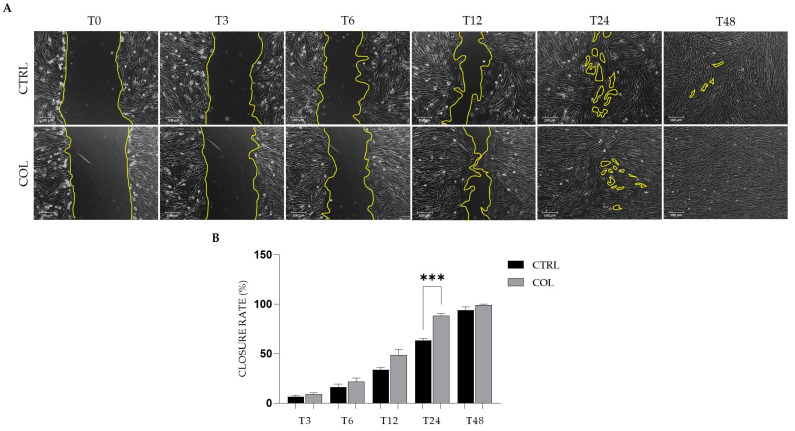
(**A**) Representative phase-contrast micrographs of hGF migration property on uncoated (CTRL) and collagen-coated (COL) cell culture plastic substrates from the time of the scratch (T0) to 48 h (T48). Images were taken by PAULA Smart Cell Imager. Scale bar: 100 µm. (**B**) Histograms representing the percentage of closure rate in the different experimental conditions. Results are expressed as mean ± standard error (n ≥ 3). Data were obtained by analyzing at least three different fields for each image with ImageJ software. (*** *p* < 0.001).

The migration ability of hGF on the collagen coating was used to study the wound healing property of these cells in vitro, using the scratch assay. Results revealed that at each time point, the wound closure occurred faster in COL hGF than in CTRL. Statistical significance was reached after 24 h when the closure rate appeared almost complete. At 48 h, a uniform monolayer was observed with no spaces among cells, indicating complete wound closure (Figure 5). Furthermore, from a morphological point of view, at 48 h, a more aligned and regular orientation in the direction of wound closure was also observable in hGF grown on the collagen coating.

### 3.4. Effect of Dental SKIN BioRegulation on FAK, YAP/TAZ Activation

As we reached promising results in wound healing, and in order to better understand whether this collagen bio-scaffold could affect hGF migration by mechanical stimulation, we analyzed the protein expression of key mechanosensors involved in cell migration.

**Figure 6:**
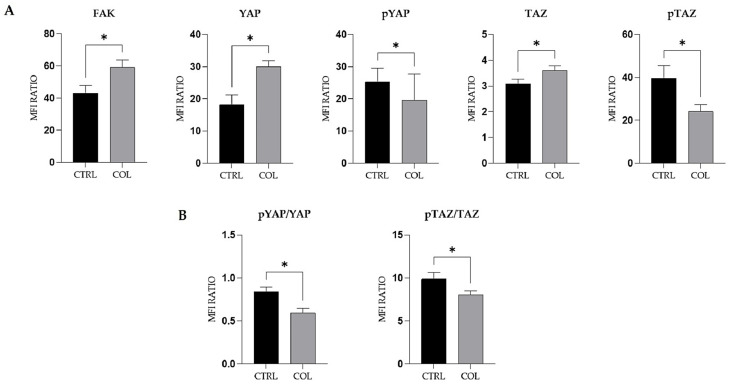
(**A**) Bar graphs of YAP and TAZ activated and inactivated (phosphorylated) forms, and (**B**) pYAP/YAP and pTAZ/TAZ ratios expressed by hGF grown on collagen-coated (COL) or uncoated cell culture plastic substrates (CTRL) after 48 h. Data are expressed as mean ± standard error (n ≥ 3). (* *p* < 0.05).

One of the first adhesion molecules recruited by cells after the ECM mechanical stimulus and designated to mechanosensory signaling is FAK. In this case, FAK appeared significantly upregulated in the presence of the collagen coating compared to controls where a mechanical stimulus was not applied (Figure 6A).

Subsequently, we evaluated another mechanism involved in cellular mechanotransduction, represented by the Hippo pathway, whose protagonists are the transcription factors YAP and TAZ. Their activity is regulated by phosphorylation and nuclear-cytoplasmic translocation. Phosphorylation inactivates YAP and TAZ, which translocate to the cytoplasm, while stiffness of the ECM affects their activation, resulting in YAP/TAZ dephosphorylation and co-translocation to the nucleus.

Interestingly, our cytometric analysis demonstrated a significant increase in individual YAP and TAZ protein expression after culturing on the collagen coating, as well as in their phosphorylated forms when hGF were cultured on uncoated cell culture substrates in the absence of a mechanical stimulus (Figure 6A). A similar pattern was observed in determining the pYAP/YAP and pTAZ/TAZ ratios, confirming the effect of the collagen coating in the activation of YAP and TAZ compared to controls (Figure 6B).

## 4. Discussion

Collagen is the most abundant protein in the body and is an essential constituent of many tissues, e.g., connective tissues, muscles, tendons, cartilage, bone, skin, and oral mucosa. It plays a functional key role in maintaining the structure of the ECM by regulating its molecular and cellular interactions and defining the shape, thickness, and resistance of the tissue. In particular, the maxillofacial region is constituted by hard and soft tissues, in which collagen represents an important component. In fact, among dental tissues, excluding enamel, collagen is found in the gingiva, dentin, cementum, pulp, and periodontal ligament [48].

Therefore, collagen is ideally suited for development as a biomedical material. As a matter of fact, in recent decades, a wide variety of collagen-based medical products of different formats and from different sources have proven to be safe and effective for various biomedical applications [33,49].

In the present study, we tested the in vitro effect of Dental SKIN BioRegulation (Guna S.p.a., Milan, Italy), an injectable medical device mainly composed of collagen type I of porcine origin, on the primary culture of hGF. Particularly, the attention was focused on cell viability, proliferation, and migration effects. In addition, since Dental SKIN BioRegulation (Guna S.p.a., Milan, Italy) was designed to act as a mechanical bio-scaffold for skin and oral soft tissues, the expression and activation of specific mechanotransduction markers were also evaluated.

The following results demonstrated that this collagen-based medical device in its coating form was not cytotoxic and increased the hGF viability, reaching statistical significance after 48 h. Interestingly, after a shorter time (24 h), it was possible to observe a completely different cell morphology compared to the CTRL grown without collagen. In particular, hGF grown on Dental SKIN BioRegulation (Guna S.p.a., Milan, Italy) appeared more closely intertwined with numerous large radial filopodia and lamellipodia-like structures than CTRL, which were more elongated and regularly arranged, suggesting that the use of this collagen coating probably resulted in an increased adhesion to the substrate.

These data were in line with previously published data, which, using the same collagen-based injectable medical device as a coating, demonstrated increased viability and substrate attachment of human tenocytes, known specialized fibroblasts of tendons [39,40]. On the contrary, other authors have found no advantages in the use of a collagen coating, demonstrating that fibroblasts (in this case human skin fibroblasts), grown on collagen-coated dishes, were rounder in shape and with a shorter perimeter, compared to those grown on uncoated polystyrene or glass culture dishes [50]. Furthermore, the number and density of cells at confluence were not affected by that specific collagen coating as well, but it should be probably considered that the fibroblast’s origin and the type of collagen used for their experimentation were different from those used in the present study and in the study conducted by Randelli et al. [40].

Despite this, there were numerous other studies that employed even more complex collagen matrices than a simple coating, confirming the positive effect of this structural protein on the growth, vitality, and migration of fibroblasts [51,52,53,54,55,56].

Regarding the latter aspect, the in vitro migration assay performed here demonstrated that Dental SKIN BioRegulation (Guna S.p.a., Milan, Italy) coating has induced a significant improvement in wound healing at 24 h and a complete reconstitution of the cell monolayer after 48 h compared to CTRL, allowing a more aligned and regular orientation of hGF in the direction of wound closure. In support of this evidence, there is a large literature on the beneficial effects of collagen in wound healing in different tissue districts, including the oral ones [56,57,58].

It is important to underline that, in addition to exogenous sources such as injectable collagen or more complex collagen matrices, there are different populations of multifunctional fibroblasts residing in the connective tissue of the gingiva and periodontal ligament able to synthesize collagen and mediate the remodeling of the developing collagen matrix. This is of great importance for the restoration of a functional periodontium [58]. In particular, during the remodeling phase, the ECM is influenced by mechanical stimuli that reflect on the population of fibroblasts. Mechanoresponsive cells play a key role in the perception of mechanical signals and in turn convert them into biological responses [58,59,60,61].

In this context, and thanks to the data obtained in this study, which demonstrated an accelerated and directional migration of hGF grown on the collagen coating, it was possible to hypothesize that the regenerative activity of this medical device was triggered by a system of mechanotransduction, as previously hypothesized [40]. Thus, to further corroborate this concept, the protein expression and activation of specific key markers involved in mechanotransduction processes, such as FAK and YAP/TAZ from the Hippo pathway, were investigated.

Interestingly, COL cultures resulted in a significant upregulation of FAK, the first protagonist in the control of cell adhesion and migration, and in a significant decrease in YAP/TAZ phosphorylation levels, corresponding to their co-translocation into the nucleus and consequent activation. More specifically, YAP/TAZ activation typically promotes a proliferative cell phenotype by inhibiting the differentiating one. These results are consistent with those obtained in previous research conducted by Randelli et al. [40], which constitutes the only study testing the identical collagen product used in the present work, with the exception of the lack of significant difference in the expression and phosphorylation of YAP/TAZ by tenocytes grown on coated or uncoated cell culture substrates, respectively. In this regard, it should be considered that those cells were different from gingival fibroblasts, and that many factors could simultaneously influence the intricate mechanism of phosphorylation and translocation of YAP/TAZ [24]. Nevertheless, immunofluorescence analysis confirmed a stronger YAP/TAZ nuclear localization in tenocytes grown on the collagen coating, as well as an upregulation of FAK mRNA levels and a significant increase in wound closure at 24 h in the same conditions.

However, the data found in this work were corroborated by numerous other studies that highlighted the main role of YAP and TAZ, belonging to the Hippo pathway, but also of FAK, in the regulation of mechanosensing and mechanotransduction of cell substrate rigidity and tensile inputs from the ECM [20,21,22,24]. Recently, the function of the Hippo signaling pathway has attracted much attention in the oral field for both craniofacial development and disease processes [62]. In particular, it has been proposed that the study of the Hippo pathway could provide a new strategy for periodontal tissue regeneration, which could represent a direction for future research [62].

Within the limitations of the present work, the lack of studies aimed at further characterizing the Hippo pathway with the expression of other markers or at investigating the COL-1 production induced by the collagen coating could be identified. Even though this research constituted a brief evidence of the morpho-functional properties of Dental SKIN BioRegulation (Guna S.p.a., Milan, Italy), the significant improvement of wound closure and the protein expression of fundamental markers involved in the cell mechanoresponsiveness to the ECM biomechanical changes suggested the effectiveness of this medical device in improving hGF focal adhesion and migration ability for regenerative purposes.

Overall, the present results seem to follow this direction and lead to the hypothesis that Dental SKIN BioRegulation (Guna S.p.a., Milan, Italy) could represent an effective oral and skin tissue regenerative approach, acting as a mechanical bio-scaffold, as it was conceived. Indeed, this medical device has been designed to mechanically direct cell migration and limit the physiological deterioration of connective tissues, giving physical and chemical support to collagen fibers present in the oral soft tissues and the subcutaneous connective tissue.

Although the effects of this type of collagen on dermal fibroblasts have not been studied yet, it was proven that these cells share 95% homology with gingival fibroblasts [63], possibly suggesting that similar mechanisms of action could also be established in the skin. Nowadays, more and more attention is arising towards minimally invasive techniques for slowing down the aging process or improving diseases related to collagen loss. In a recent literature review, several minimally invasive surgery approaches in the improvement of facial and perioral aging were summarized, including the use of collagen [64]. For this reason, alongside the need for further studies in the dental field, it could also be interesting to investigate the beneficial effects of this injectable collagen on dermal fibroblasts to broaden its application as a dental, perioral, and facial rejuvenation treatment.

Collagen can be used as an innovative, therapeutic, regenerative, and rehabilitative approach to promote soft tissue healing and maintenance through infiltrative techniques, as well as an effective dental implant coating, as described in this context. Recent literature has described the immobilization of type I collagen on implant surfaces as a means to improve fibroblast response and to accelerate the healing processes of the gingival tissue, although a functionalized implant surfaces has not yet been launched on the market [35,65,66].

In order to take advantage of all its potential application forms, collagen-modified titanium implant surfaces could be developed to support the maintenance of peri-implant soft tissues health, ensuring bacterial seal and preventing bacterial colonization and the onset of peri-implantitis. Additionally, investigating and evaluating other potential effects of Dental SKIN BioRegulation (Guna S.p.a., Milan, Italy) on jaw-bone-derived osteoblasts could serve as a promising tool for improving the long-term bony and soft tissue integration of dental implants.

## 5. Conclusions

Overall, this study sheds light on the possible use of a new non-invasive, safe, and effective injectable medical device based on collagen, which could be proposed as a valid treatment for regeneration therapies. Specifically, based on these findings, Dental SKIN BioRegulation (Guna S.p.a., Milan, Italy) could represent a useful treatment in gingival rejuvenation and may possibly be helpful in the resolution of oral pathologies characterized by the structural deterioration of the connective tissue, such as periodontitis and peri-implantitis.

In conclusion, the present research adds knowledge to a large and controversial scenario of oral therapies aimed at preserving the health of periodontal and peri-implant soft tissues to maintain the long-term health of teeth and dental implants.

## Data Availability

All experimental data to support the findings of this study are available by contacting the corresponding author upon request.
